# Identifying Conserved Regions in HIV-1 Proteins by Entropy Analysis of Sequence Variability

**DOI:** 10.3390/ijms27115139

**Published:** 2026-06-05

**Authors:** Alexandr N. Shchemelev, Elena N. Serikova, Yulia V. Ostankova, Vladimir S. Davydenko, Edward S. Ramsay, Areg A. Totolian

**Affiliations:** Saint Petersburg Pasteur Institute, 197101 St. Petersburg, Russia; genista.bio@gmail.com (E.N.S.); shenna1@yandex.ru (Y.V.O.); vladimir_david@mail.ru (V.S.D.); totolian@spbraaci.ru (A.A.T.)

**Keywords:** HIV-1, genetic diversity, sequence conservation, Shannon entropy, gag, pol, env, accessory proteins, conserved regions, bioinformatics

## Abstract

The extraordinary genetic diversity of human immunodeficiency virus type 1 (HIV-1), driven by high mutation and recombination rates, poses significant challenges for diagnostics, therapy, and vaccine development. While variable regions enable immune escape, hyperconserved regions are critical for viral function and represent promising targets for novel therapeutic interventions. This study aimed to develop and validate a bioinformatic algorithm for quantitative assessment of sequence conservation and automated identification of functionally significant conserved regions across all major HIV-1 proteins. A total of 1119 full-length HIV-1 genome sequences representing major subtypes (A1, A2, A6, B, C, D, F1, F2, G, H, J, K) were analyzed. Normalized Shannon entropy (S-index) was calculated for each alignment column. Statistical thresholds for conserved regions were established using 95% confidence intervals derived from bootstrap resampling. Two complementary algorithms, clustering and local maxima detection, were applied to identify conserved regions, which were subsequently mapped to known functional domains based on literature data. Protein conservation varied markedly, with S_m_ values ranging from 0.784 (Vpu) to 0.920 (Pol). Gag, Pol, and Vpr demonstrated the highest overall conservation, while Env, Rev, Tat, and Vpu exhibited pronounced variability interspersed with conserved domains. In total, 25 conserved regions in Gag, 49 in Pol, 28 in Env, and 6–4 regions in accessory proteins (Vif, Vpr, Rev, Tat, Nef, Vpu) were identified. These regions corresponded to critical functional elements including enzyme catalytic centers, zinc fingers, receptor-binding sites, protein interaction interfaces, and membrane-anchoring domains. The developed computational framework enables statistically grounded identification of evolutionarily constrained regions across analyzed HIV-1 subtypes. The identified conserved regions represent candidate sites for further investigation and may inform downstream studies focused on antiviral target prioritization, immunogen design, and diagnostic assay development. However, their translational applicability requires additional analytical, structural, and experimental validation.

## 1. Introduction

### 1.1. Global HIV Variability

The human immunodeficiency virus (HIV) remains one of the most serious global public health challenges. According to data from the World Health Organization (WHO), as of the end of 2024, approximately 40.8 million people worldwide were living with HIV, and 1.3 million new infections were reported [1]. One of the key characteristics that underlies the complexity of controlling this infection is the extraordinary genetic diversity of the virus, which exhibits distinct geographic patterns.

The virus is classified into two main types: HIV-1, which accounts for the overwhelming majority of infections globally, and HIV-2, which is less virulent and is predominantly distributed in West Africa. HIV-1 has the greatest epidemiological significance and is further divided into several groups. Group M (“major”) is responsible for more than 90% of all infections and comprises multiple subtypes (A, B, C, D, F, G, H, J, K) as well as circulating recombinant forms (CRFs), which differ from one another by approximately 25–35% at the nucleotide sequence level. Subtype B has historically predominated in Europe, North America, and Australia. Subtype C is widespread in Southern Africa and India. Subtype A and recombinant forms derived from it, such as CRF02_AG, circulate in West and East Africa [2], as well as in countries of Eastern Europe and Central Asia [3].

The Russian Federation provides an illustrative example of changes in the landscape of circulating HIV-1 variants. Whereas in 2008–2010, subtype A accounted for 91.3% of cases and subtype B for 8.7%, during 2011–2014 the spectrum of genetic diversity expanded due to the emergence of recombinant forms (AB, AG, CRF06_cpx) and subtype C [4,5,6]. This genetic diversity affects diagnostic accuracy, therapeutic efficacy, and vaccine development strategies, rendering global surveillance of HIV strains critically important.

### 1.2. Biological Nature of HIV Variability

The fundamental basis of the high variability of HIV lies in the characteristics of its replicative cycle as a retrovirus. A key role is played by the enzyme reverse transcriptase, which synthesizes DNA using viral RNA as a template. This enzyme lacks proofreading and error-correction mechanisms, resulting in the frequent occurrence of mutations at a rate of (4.1 ± 1.7) × 10^−3^ per nucleotide, which is the highest value reported for any biological entity [7]. More than one billion new viral particles are produced daily in an infected organism; this intensity, combined with the high mutation rate, generates an enormous number of genetic variants [8].

This process is further exacerbated by the virus’s capacity for recombination. When a single cell is infected by two different HIV strains, their genetic material can “mix” during the assembly of new virions, leading to the emergence of CRFs that may possess novel properties [9]. Natural selection exerted by the human immune system and, critically, by antiretroviral therapy (ART) favors mutant variants capable of evading immune control or exhibiting resistance to antiretroviral drugs.

Of particular interest is the interaction between the virus and host chemokine receptors. The majority of primary infections are caused by viral variants (R5) that utilize the CCR5 receptor. Human genetic polymorphisms, such as the well-characterized Δ32 deletion in the CCR5 gene, result in the production of a defective receptor and may confer resistance to HIV infection [10]. Other mutations, for example in the CCR2 or CXCL12 genes, are associated with delayed disease progression.

### 1.3. Methods of Genetic Variability Research

Contemporary analyses of the genetic variability of HIV-1 are based on integrated bioinformatic approaches that enable investigation of viral evolution at multiple structural and functional levels. Phylogenetic analysis remains a fundamental tool for reconstructing the evolutionary history of viral isolates and tracing transmission pathways. With the advent of next-generation sequencing (NGS) technologies, it has become possible to study not individual strains, but the entire spectrum of HIV quasispecies within a single patient, which is critically important for understanding the mechanisms underlying the development of drug resistance [11]. However, a mere description of genetic diversity has proven insufficient for addressing applied problems in antiviral therapy.

To identify functionally significant regions of the viral genome, analyses of evolutionary conservation are widely employed based on the calculation of Shannon entropy or similar metrics of nucleotide sequence variability [12,13]. Hyperconserved regions, which exhibit minimal variability even among different HIV-1 subtypes, are considered promising targets for the development of novel therapeutic agents as their stability indicates critical functional importance for the viral life cycle. In particular, the structural proteins capsid p24 and nucleocapsid p7 demonstrate a high degree of conservation, which explains their attractiveness as targets for new classes of drugs, such as capsid inhibitors [14].

Studies have shown that even highly conserved regions, such as the 5′ untranslated region (5′ UTR), display substantial interstrain variability, up to 17% in the U5–PBS region and up to 20% in the gag leader sequence (GLS), among different subtypes and [15]. This variability may create strain-specific transcription factor binding sites (for example, an E-box in CRF22_01A1 or Stat6 in subtypes A and G), potentially affecting the efficiency of viral replication. Hyperconserved regions exhibiting minimal variability even between different HIV-1 subtypes are therefore regarded as promising targets for the development of new therapeutic strategies, as their stability reflects their critical functional role in the viral life cycle.

Of particular importance is the analysis of covariation networks of amino acid residues, which enables the identification of compensatory mutations arising in response to the selective pressure exerted by antiretroviral therapy. For example, mutations conferring resistance to reverse transcriptase inhibitors are often accompanied by specific changes in other regions of the protein that restore the functional activity of the enzyme. Modern algorithms, such as methods for detecting coevolving positions (e.g., Direct Coupling Analysis), allow prediction of the emergence of such compensatory mutations and may be used to optimize antiretroviral treatment regimens.

Molecular modeling and prediction of the three-dimensional structures of viral proteins harboring resistance-associated mutations make it possible to interpret observed genetic changes at the structural level. Machine learning-based approaches are increasingly applied for the classification of viral subtypes, prediction of antiretroviral drug resistance, and identification of novel potential therapeutic targets. Nevertheless, despite the abundance of bioinformatic methods, there remains a lack of standardized solutions for comprehensive screening of large sequence datasets with simultaneous assessment of conservation, variability, and functional relevance of identified regions. The development of integrated algorithms combining phylogenetic analysis, evaluation of evolutionary pressure, and prediction of the structural and functional consequences of mutations represents a pressing challenge in HIV bioinformatics.

Thus, the aim of the present study was to develop an algorithm for the screening and analysis of nucleotide and amino acid sequence datasets that enables quantitative assessment of their conservation and variability, as well as automated identification of hyperconserved regions with potentially critical functional significance.

## 2. Results

### 2.1. Determination of Conservation Parameters for Sequence Alignment and Establishment of Cutoff Thresholds

**Figure 1:**
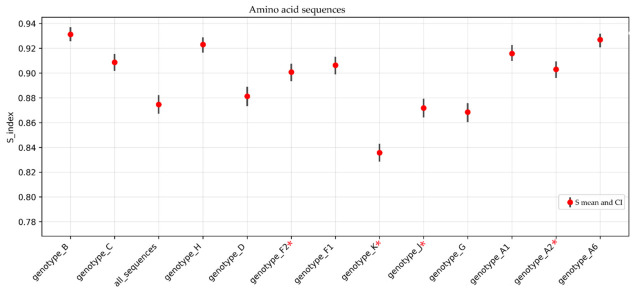
Aggregate values for amino acid sequence alignments of different HIV-1 subtypes. Red asterisks indicate subtypes represented by a limited number of sequences in the alignment.

Calculation results for amino acid sequence alignments are presented in Figure 1 and Table 1. Among the sufficiently well-represented HIV-1 subtypes (i.e., those for which more than 20 sequences were present in the alignment), subtypes D, F1, and G exhibited higher levels of diversity, whereas subtypes A6, B, and H were the most conserved. For several genotypes (A2, F2, J, K), insufficient numbers of sequences meeting the inclusion criteria were available to allow an unambiguous assessment of their degree of conservation. Nevertheless, cutoff thresholds for the identification of highly conserved and highly variable regions were also calculated for these genotypes for subsequent analyses. The confidence intervals presented in Table 1 were adopted as the cutoff thresholds.

### 2.2. Determination of Viral Protein Amino Acid Homogeneity and Identification of Region Type (Conserved, Variable) Within Sequence Alignments

#### 2.2.1. Group-Specific Antigen

**Figure 2:**
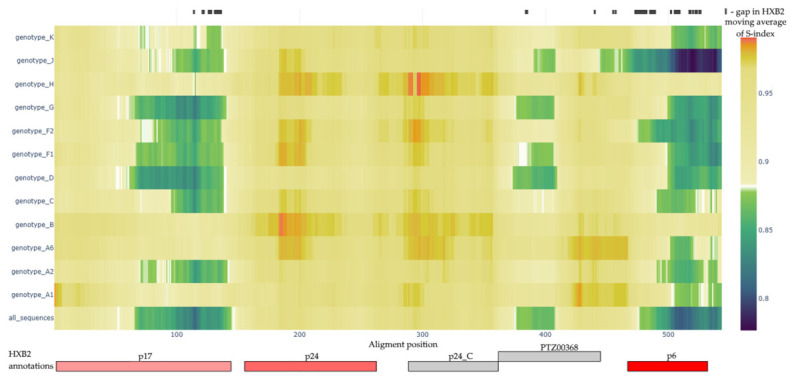
Averaged S-index panorama for the alignment of Gag amino acid sequences obtained using a sliding window of 50 amino acids. The colors correspond to the mean S-index value according to the color scale to the right of the figure. The marks above the figure indicate MSA regions corresponding to gaps in the HXB2 sequence. Below the figure, annotations of the HXB2 sequence are shown in alignment coordinates, with colors chosen randomly for contrast.

For amino acid sequence alignments of the group-specific antigen (Gag) polyprotein, an analysis was performed to examine the dynamics of conservation, and averaged S-index panoramas were obtained (Figure 2), along with generalized statistics describing the conservation of this region (Table 2).

**Figure 3:**
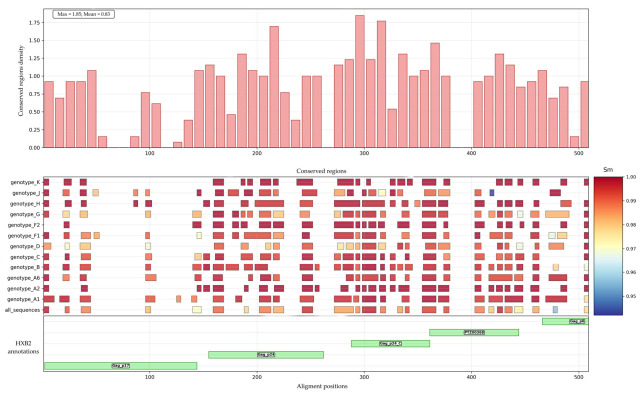
Generalized representation of conserved regions identified in Gag amino acid sequence alignments. The colors correspond to the mean S-index value according to the color scale to the right of the figure. Below the figure, annotations of the HXB2 sequence are shown in alignment coordinates.

Gag was shown to be a relatively conserved region with distinct variable segments. Clearly defined conserved regions can be observed in amino acid sequences, and these regions largely coincide with one another. Both conserved and variable regions were identified within the alignments, and the summarized results of this analysis for the protein alignments are presented in Figure 3 and Table 3.

Across alignments of different HIV-1 subtypes, 23–30 regions with S_m_ values ranging from 0.9856 to 1.0000 were identified. Alignment of all sequences made it possible to detect 26 conserved regions with an S_m_ value of 0.9856. In addition, the alignments were analyzed using an algorithm based on the calculation of local maxima. The results of this supplementary analysis, together with filtering of the identified conserved regions whose consensus sequences consisted predominantly of gaps, allowed the identification of 25 principal conserved regions of Gag, which are presented in Table 4.

#### 2.2.2. Polymerase

**Figure 4:**
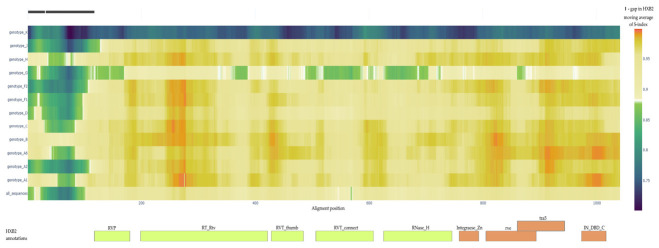
Averaged S-index panorama for the alignment of Pol amino acid sequences obtained using a sliding window of 50 amino acids. The colors correspond to the mean S-index value according to the color scale to the right of the figure. The marks above the figure indicate MSA regions corresponding to gaps in the HXB2 sequence. Below the figure, annotations of the HXB2 sequence are shown in alignment coordinates, with colors chosen randomly for contrast.

The polymerase (Pol) polyprotein as a whole demonstrates a uniformly high level of conservation throughout its entire length. The averaged panorama for the aggregate of all subtypes does not reveal clearly pronounced peaks of conservation or variability, indicating relatively homogeneous evolutionary preservation of this protein (Figure 4, Table 5). However, alignments of individual subtypes do exhibit distinctly defined regions with increased conservation, while subtypes K and G display anomalously low levels of amino acid homogeneity. For subtype K, this may be explained by the limited number of sequences included in the alignment. However, the result obtained for subtype G remains anomalous and is likely attributable to an algorithmic artifact.

**Figure 5:**
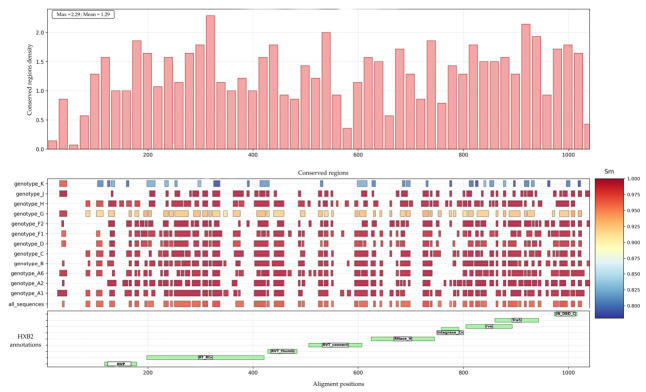
Generalized representation of conserved regions identified in Pol amino acid sequence alignments. The colors correspond to the mean S-index value according to the color scale to the right of the figure. Below the figure, annotations of the HXB2 sequence are shown in alignment coordinates.

Although the Pol region proved to be relatively uniformly conserved along its entire length, cluster analysis enabled the identification of 44 to 54 conserved regions depending on subtype (excluding subtype K), with Sm values ranging from 0.92775 to 1.0000 (Figure 5, Table 6).

Supplementary Analysis using the local maxima algorithm, followed by final filtering, enabled the identification of 49 major conserved regions within Pol, which are presented in Table 7.

#### 2.2.3. Envelope

**Figure 6:**
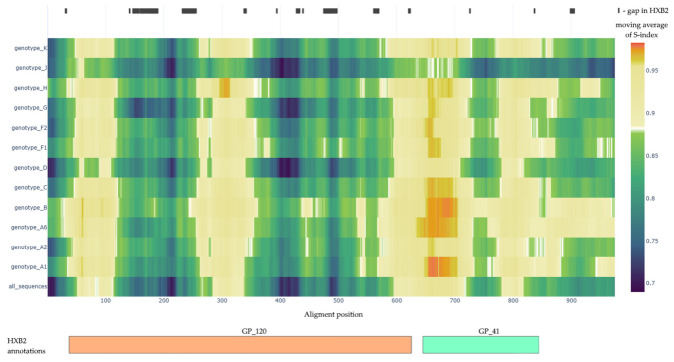
Averaged S-index panorama for the alignment of Env amino acid sequences obtained using a sliding window of 50 amino acids. The colors correspond to the mean S-index value according to the color scale to the right of the figure. The marks above the figure indicate MSA regions corresponding to gaps in the HXB2 sequence. Below the figure, annotations of the HXB2 sequence are shown in alignment coordinates, with colors chosen randomly for contrast.

In contrast to the more conserved Gag and Pol polyproteins, envelope (Env) exhibits the highest level of variability. The averaged S-index panoramas obtained from amino acid sequence alignments (Figure 6) and the low conservation index values (Table 8) indicate pronounced heterogeneity in this region.

**Figure 7:**
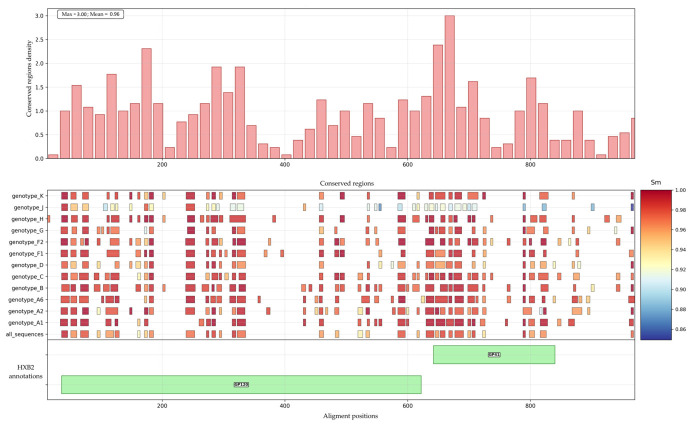
Generalized representation of conserved regions identified in Env amino acid sequence alignments. The colors correspond to the mean S-index value according to the color scale to the right of the figure. Below the figure, annotations of the HXB2 sequence are shown in alignment coordinates.

For systematic identification of conserved elements, a clustering algorithm was first applied, which detected between 31 and 41 such regions depending on subtype. These identified regions exhibited high internal conservation (S_m_ ranging from 0.919 to 0.988; Figure 7, Table 9).

In the second stage, using a local maxima algorithm followed by filtering, the obtained list was refined and reduced to 28 major conserved regions common to all analyzed Env sequences. The final data, including the coordinates of these regions, are presented in Table 10.

#### 2.2.4. Viral Infectivity Factor

**Figure 8:**
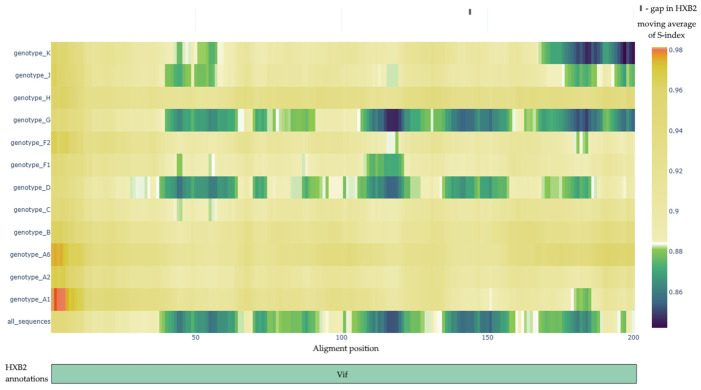
Averaged S-index panorama for the alignment of Vif amino acid sequences obtained using a sliding window of 50 amino acids. The colors correspond to the mean S-index value according to the color scale to the right of the figure. The marks above the figure indicate MSA regions corresponding to gaps in the HXB2 sequence. Below the figure, annotations of the HXB2 sequence are shown in alignment coordinates, with colors chosen randomly for contrast.

Initial analysis of alignment containing sequences of HIV viral infectivity factor (Vif) from all subtypes revealed relatively low S-index values (Figure 8, Table 11), indicating the heterogeneity of this protein across the viral population as a whole. However, analysis of subtype-stratified alignments revealed a different pattern: within each subtype, Vif exhibits a high degree of conservation. This suggests the presence of significant inter-subtype differences alongside tight stabilization of the protein within distinct viral evolutionary lineages.

**Figure 9:**
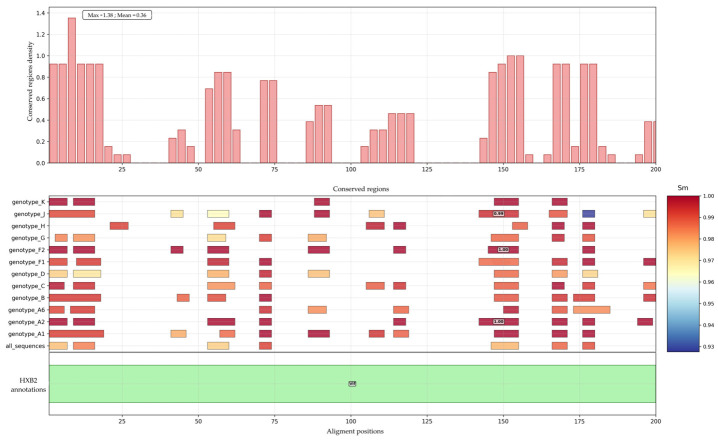
Generalized representation of conserved regions identified in Vif amino acid sequence alignments. The colors correspond to the mean S-index value according to the color scale to the right of the figure. Below the figure, annotations of the HXB2 sequence are shown in alignment coordinates.

In the next stage, using a clustering algorithm, between 5 and 10 conserved regions were identified in the individual subtype alignments, characterized by high S-index values (0.978–1.000; Figure 9, Table 12).

The final stage of analysis, employing a local maxima algorithm, enabled the consolidation and refinement of these data, identifying six major conserved regions common to all investigated HIV-1 subtypes. Detailed information on these regions, including their coordinates and Sm-index values, is provided in Table 13.

#### 2.2.5. Viral Protein R

**Figure 10:**
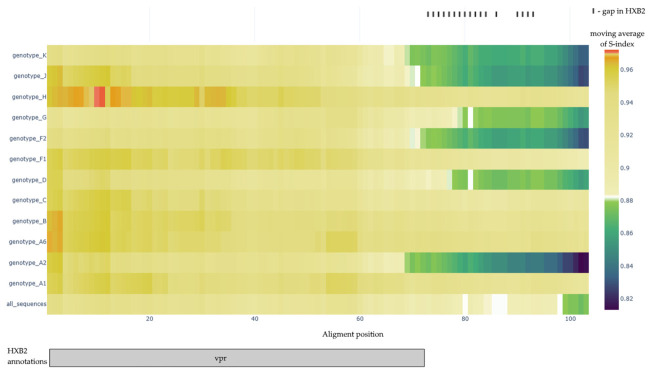
Averaged S-index panorama for the alignment of Vpr amino acid sequences obtained using a sliding window of 50 amino acids. The colors correspond to the mean S-index value according to the color scale to the right of the figure. The marks above the figure indicate MSA regions corresponding to gaps in the HXB2 sequence. Below the figure, annotations of the HXB2 sequence are shown in alignment coordinates, with colors chosen randomly for contrast.

Averaged S-index panoramas (Figure 10) for HIV viral protein R (Vpr) alignments demonstrated uniformly high values of the homogeneity index across most of the protein length. The summarized statistical data presented in Table 14 confirm this pattern: S_m_-index values for individual subtypes range from 0.902 to 0.944, indicating a relatively high degree of overall Vpr conservation.

**Figure 11:**
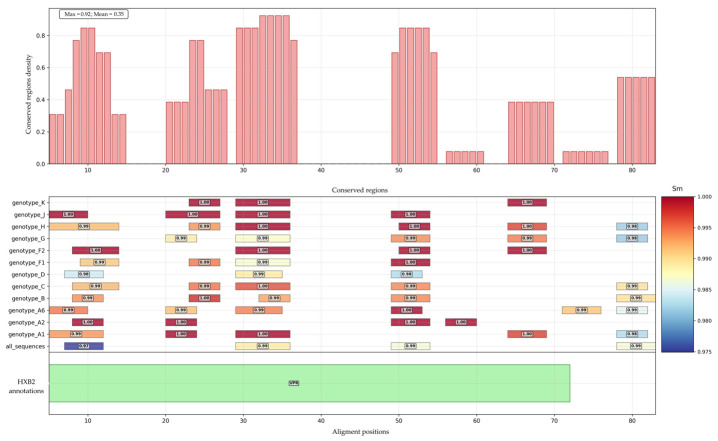
Generalized representation of conserved regions identified in Vpr amino acid sequence alignments. The colors correspond to the mean S-index value according to the color scale to the right of the figure. Below the figure, annotations of the HXB2 sequence are shown in alignment coordinates.

To identify localized regions with the highest degree of conservation, a clustering algorithm was applied. Analysis of subtype-stratified alignments enabled the identification of 3 to 6 highly conserved regions, depending on subtype. These regions were characterized by high S_m_ values ranging from 0.984 to 1.000 (Figure 11, Table 15), indicating minimal variability at the positions they encompass.

In the final stage, a local maxima algorithm was used to refine the boundaries of the conserved regions and eliminate redundant fragments. This approach enabled the consolidation and filtering of data obtained for different subtypes, leading to the identification of four major conserved regions common to all investigated HIV-1 subtypes. Detailed information on each region, including coordinates relative to the HXB2 reference sequence, as well as the alignment, length, and S_m_ values, is provided in Table 16.

#### 2.2.6. Regulator of Virion Expression

**Figure 12:**
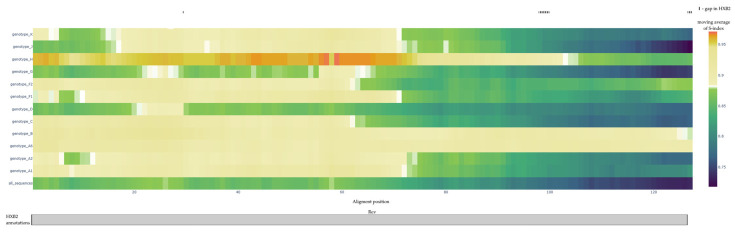
Averaged S-index panorama for the alignment of Rev amino acid sequences obtained using a sliding window of 50 amino acids. The colors correspond to the mean S-index value according to the color scale to the right of the figure. The marks above the figure indicate MSA regions corresponding to gaps in the HXB2 sequence. Below the figure, annotations of the HXB2 sequence are shown in alignment coordinates, with colors chosen randomly for contrast.

In the first stage of analysis, the averaged S-index panoramas for regulator of virion expression (Rev) sequences alignments revealed a non-uniform conservation profile: against a background of overall variability, several distinct regions with elevated homogeneity index values were clearly distinguishable (Figure 12, Table 17). The S_m_ value for the alignment of all subtypes was 0.825, while for individual subtypes this value ranged from 0.836 (subtype D) to 0.930 (subtype H), indicating substantial inter-subtype differences in the degree of Rev conservation.

**Figure 13:**
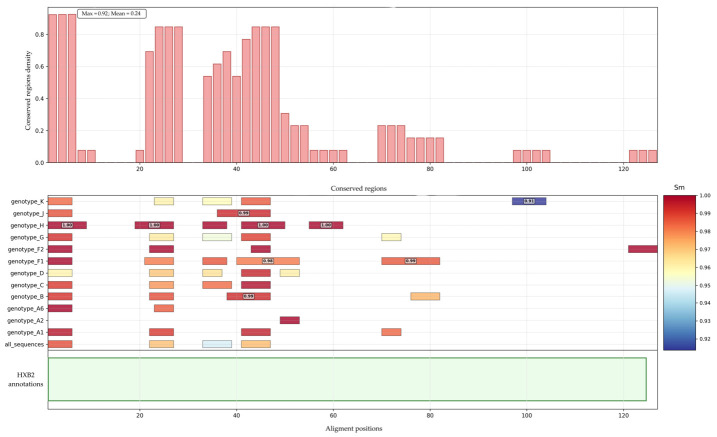
Generalized representation of conserved regions identified in Rev amino acid sequence alignments. The colors correspond to the mean S-index value according to the color scale to the right of the figure. Below the figure, annotations of the HXB2 sequence are shown in alignment coordinates.

To identify localized regions with the highest degree of conservation, a clustering algorithm was applied at the second stage (Figure 13). Analysis of subtype-stratified alignments enabled the identification of 1 to 5 highly conserved regions, depending on subtype. These regions were characterized by high S_m_ values ranging from 0.9600 to 1.000 (Table 18), indicating minimal variability at the positions they encompass. The smallest number of conserved regions (1–2) was detected for subtypes A2 and J, which is likely associated with the most uniform sequence conservation, whereas for most other subtypes, their number ranged from 4 to 5.

In the final stage, a local maxima algorithm was used to refine the boundaries of the conserved regions and eliminate fragmented segments. This approach enabled the consolidation and filtering of data obtained for different subtypes, leading to the identification of four major conserved regions common to all investigated HIV-1 subtypes. Detailed information on each region, including coordinates relative to the HXB2 reference sequence, as well as the alignment, length, and S_m_ values, is provided in Table 19.

#### 2.2.7. Trans-Activator of Transcription

**Figure 14:**
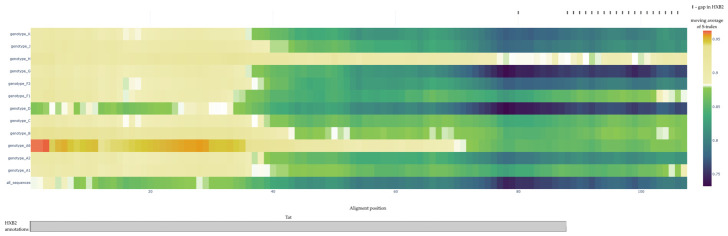
Averaged S-index panorama for the alignment of Tat amino acid sequences obtained using a sliding window of 50 amino acids. The colors correspond to the mean S-index value according to the color scale to the right of the figure. The marks above the figure indicate MSA regions corresponding to gaps in the HXB2 sequence. Below the figure, annotations of the HXB2 sequence are shown in alignment coordinates, with colors chosen randomly for contrast.

The averaged S-index panorama for alignments of trans-activator of transcription (Tat) sequences obtained at the first stage of analysis revealed a pronounced non-uniformity in the conservation profile (Figure 14). The N-terminal region of the protein is characterized by relatively high homogeneity index values. The remainder of the sequence exhibits significantly higher variability. The summarized statistical data presented in Table 20 confirm this overall pattern: the S_m_ value for the alignment of all subtypes was 0.8205. When analyzing individual subtypes, this value ranged from 0.818 (subtype D) to 0.905 (subtypes A6, H), indicating substantial differences in Tat between sequences of different subtypes.

**Figure 15:**
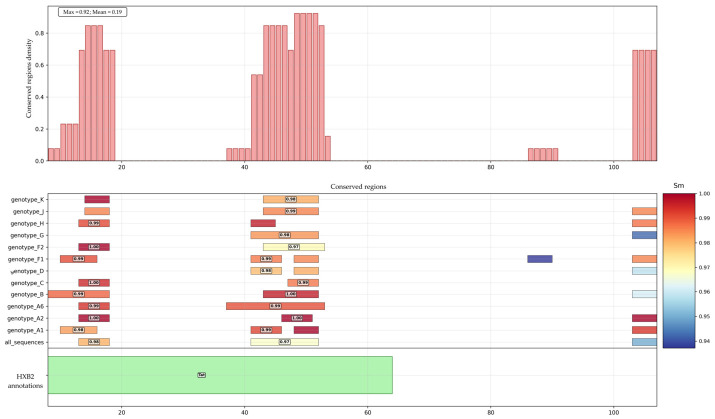
Generalized representation of conserved regions identified in Tat amino acid sequence alignments. The colors correspond to the mean S-index value according to the color scale to the right of the figure. Below the figure, annotations of the HXB2 sequence are shown in alignment coordinates.

To identify localized regions with the highest degree of conservation, a clustering algorithm was applied at the second stage. The analysis enabled the identification of 2 to 5 highly conserved regions, depending on subtype (Figure 15). These regions were characterized by high S_m_ values ranging from 0.963 to 1.000 (Table 21), indicating minimal variability at the positions they encompass. The smallest number of conserved regions (2) was detected for subtypes A6, C, F2, G, and K. For subtype F1, it reached 5.

In the final stage of analysis, two major conserved regions common to all investigated HIV-1 subtypes were identified. Detailed information on both regions, including coordinates relative to the HXB2 reference sequence, as well as the alignment, length, and S_m_ values, is provided in Table 22.

#### 2.2.8. Negative Regulatory Factor

**Figure 16:**
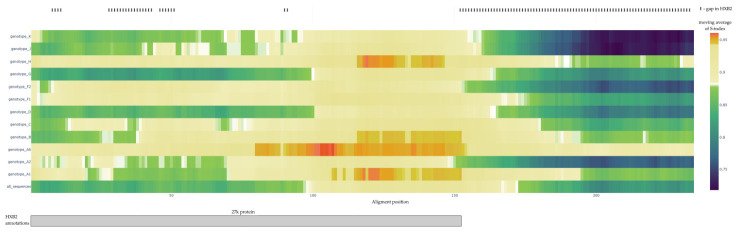
Averaged S-index panorama for the alignment of Nef amino acid sequences obtained using a sliding window of 50 amino acids. The colors correspond to the mean S-index value according to the color scale to the right of the figure. The marks above the figure indicate MSA regions corresponding to gaps in the HXB2 sequence. Below the figure, annotations of the HXB2 sequence are shown in alignment coordinates, with colors chosen randomly for contrast.

Sequence analysis revealed that negative regulatory factor (Nef) is characterized by a pronounced non-uniform distribution of conserved and variable regions (Figure 16). The averaged S-index panoramas display clearly defined peaks of conservation in the N-terminal region and several internal domains, whereas a substantial portion of the sequence exhibits increased variability. According to the summarized statistical data (Table 23), the Sm-index value for the alignment of all subtypes was 0.866. When examining individual subtypes, this value varies over a wide range: from 0.847 (subtype K) to 0.926 (subtype A6).

**Figure 17:**
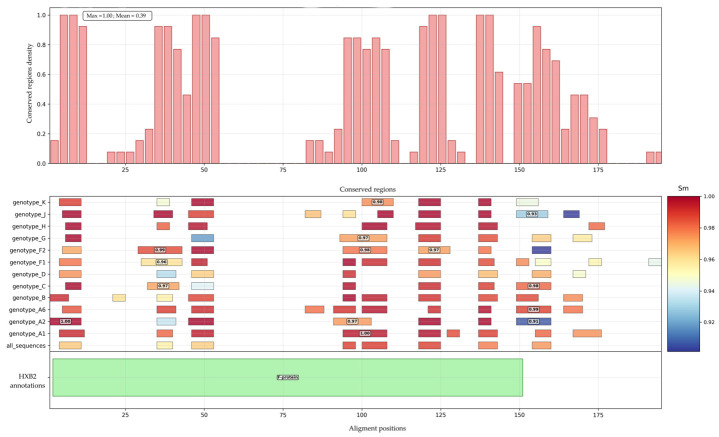
Generalized representation of conserved regions identified in Nef amino acid sequence alignments. The colors correspond to the mean S-index value according to the color scale to the right of the figure. Below the figure, annotations of the HXB2 sequence are shown in alignment coordinates.

This protein proved to be relatively highly variable; however, its N-terminus demonstrated high conservation within subtypes. Depending on the subtype, between 7 and 11 highly conserved regions were identified (Figure 17, Table 24). The identified regions exhibit high S_m_ values ranging from 0.966 to 0.994, indicating extremely low variability at the amino acid positions they encompass. The highest number of conserved regions (10–11) was characteristic of subtypes A6, B, F1, and J. For subtypes A2, C, F2, G, H, and K, their number ranged from 7 to 8.

In the final stage, using a local maxima algorithm followed by the filtering of data obtained for different subtypes, six major conserved regions common to all investigated HIV-1 subtypes were identified. General information on these regions, including coordinates relative to the HXB2 reference sequence, as well as the alignment, length, and S_m_ values, is provided in Table 25.

#### 2.2.9. Viral Protein U

**Figure 18:**
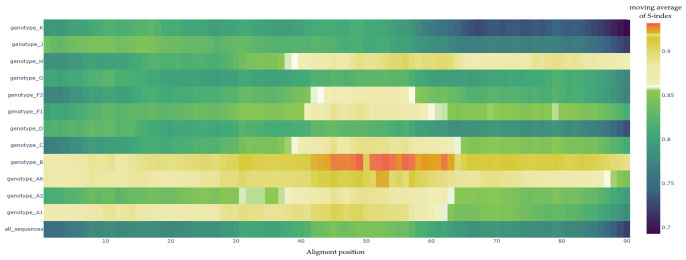
Averaged S-index panorama for the alignment of Vpu amino acid sequences obtained using a sliding window of 50 amino acids. The colors correspond to the mean S-index value according to the color scale to the right of the figure. The marks above the figure indicate MSA regions corresponding to gaps in the HXB2 sequence. Below the figure, annotations of the HXB2 sequence are shown in alignment coordinates, with colors chosen randomly for contrast.

According to data obtained in the first stage of analysis of viral protein U (Vpu) sequences and the averaged S-index diagram (Figure 18, Table 26), Vpu exhibits the lowest conservation values among all HIV-1 proteins investigated. The S_m_ value for the alignment of all subtypes combined was 0.784, indicating high variability of this protein across the viral population. Nevertheless, it can be noted that the central regions of Vpu contain a relatively conserved area, which is particularly pronounced within individual subtypes. When analyzing specific subtypes, this value varies over a wide range: from 0.773 (subtype K) to 0.905 (subtype B). The highest homogeneity index values are observed for subtypes B (0.905) and A6 (0.886). Subtypes D, G, J, and K are characterized by the lowest values (0.773–0.803), reflecting substantial inter-subtype differences in the degree of Vpu conservation.

**Figure 19:**
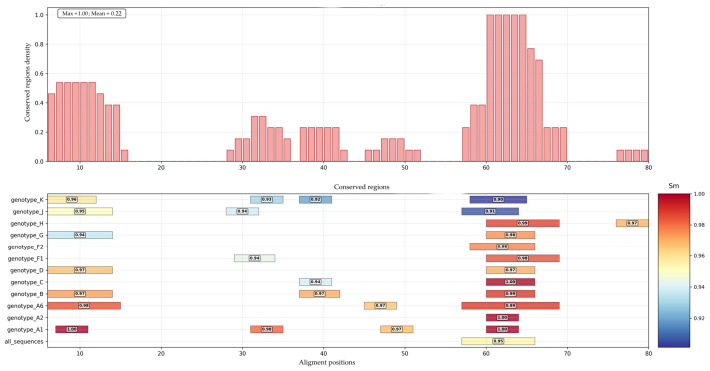
Generalized representation of conserved regions identified in Vpu amino acid sequence alignments. The colors correspond to the mean S-index value according to the color scale to the right of the figure. Below the figure, annotations of the HXB2 sequence are shown in alignment coordinates.

The applied clustering algorithm enabled the identification of 1 to 4 highly conserved regions, depending on subtype (Figure 19, Table 27). The identified regions were characterized by S_m_ values ranging from 0.927 to 1.000. The highest number of conserved regions was detected for subtypes A1 and K. For subtypes A2, F2, and for all subtypes combined (all_sequences), only one conserved region was found. The boundaries of this region were confirmed at the final stage of analysis. It is located within alignment coordinates 57–66 and spans 10 amino acid positions. The S_m_ value for this region is 0.954, indicating a high degree of conservation in this area, despite the overall variability of the Vpu protein as a whole.

## 3. Discussion

### 3.1. General Characteristics of the Obtained Alignments

The performed analysis of HIV-1 protein sequences provided a comprehensive overview of the distribution of conserved and variable regions across the major viral proteins. The applied computational approach, based on normalized Shannon entropy combined with clustering and local maxima algorithms, enabled quantitative assessment of sequence homogeneity and systematic identification of highly conserved regions potentially associated with critical viral functions.

The analyzed datasets differed substantially in size depending on subtype and genomic region. The largest number of sequences was available for globally prevalent subtypes, whereas several subtypes were represented by comparatively limited numbers of full-length genomes. This factor should be considered when interpreting the results, particularly for low-representation subtypes, in which the observed conservation may partially reflect limited sampling. Nevertheless, inclusion of these subtypes allowed a broader overview of HIV-1 diversity within Group M.

The use of a unified statistical framework based on entropy analysis enabled direct comparison of conservation profiles across proteins with distinct evolutionary characteristics. Although this approach does not fully account for differences in selective pressures between proteins, the identified conserved regions demonstrated strong correspondence with functionally important domains previously described in structural and biochemical studies, supporting the biological relevance of the obtained results.

Although the present study is purely computational and does not include experimental validation, the identified conserved regions may be relevant for future research aimed at HIV control and potential eradication strategies. Long-term viral persistence remains one of the principal barriers to HIV cure efforts, and highly conserved viral regions represent attractive candidates for approaches intended to minimize immune escape and resistance-associated variability. In particular, conserved elements identified within Gag, Pol, and structurally constrained regions of Env may support downstream development of broad-reactive immunogens, antiviral targets, or molecular diagnostic strategies intended to maintain effectiveness across genetically diverse HIV-1 variants.

In the following sections, an attempt is made to relate the identified conserved regions to functional and structural elements of HIV-1 proteins previously described in the literature, including catalytic centers, intermolecular interaction interfaces, membrane-associated domains, and other regions subject to strong evolutionary constraints.

### 3.2. Characterization of the Conserved Amino Acid Regions Identified

#### 3.2.1. Group-Specific Antigen

Gag (pr55) is synthesized in the cytoplasm as a multidomain protein with a molecular mass of 55 kDa. Gag consists of four major structural domains (from N- to C-terminus): MA (matrix), CA, NC (nucleocapsid), and p6. There are also two small peptides, spacer peptide 1 (SP1) and spacer peptide 2 (SP2), which link the CA-NC and NC-p6 domains, respectively. Historically, the understanding of the structure, maturation, and function of Gag has undergone changes. Consequently, the original annotations of the HXB2 reference sequence are partially outdated and do not fully align with the current understanding of this polyprotein’s organization. In the following text, we will endeavor to rely on contemporary insights into the arrangement of regions within this and subsequent proteins, guided by the coordinates of the obtained alignment and accompanying them with HXB2 coordinates in parentheses.

Synthesis of the analysis performed for different subtypes allowed the identification of 24 highly conserved regions within the Gag protein sequence (Table 4). These conserved regions are represented in all final protein products resulting from Gag processing and, as expected, largely correspond to known functional areas of these products.

**Matrix Protein**

Within Gag, the matrix protein is represented by the MA domain, which in our alignment corresponds to coordinates 1–143 (HXB2: 1–131). Upon final maturation, it becomes the p17 protein. This structural protein typically consists of 132 amino acids and is encoded by the *Gag* gene, playing an important role in most stages of the HIV-1 life cycle. The protein is partially globular, comprising four helices that form a dense central domain, capped by a β-sheet containing basic amino acids.

Region CR_1 corresponds to the beginning of the peptide and is located at the very start of the N-terminal domain, which, due to subsequent active myristoylation, performs the function of attaching the capsid to the lipoprotein envelope of the future virion. This same connection requires the highly basic region (HBR), within which the identified conserved region CR_2 is located. This region is rich in arginine and lysine residues and electrostatically interacts with negatively charged phospholipids located in the inner leaflet of the plasma membrane [16].

Region CR_3 is located entirely within α-helix A and largely corresponds to its boundaries. A 1997 study described the role of this helix in the interaction of p17 with the cell membrane and noted that virions with mutations in this region lacked infectious activity [17]. However, the full functional significance of this highly conserved region has not been completely elucidated. Regions CR_4 and CR_5 (partially) are located within α-helix D, whose role in p17 function has not been revealed. Nevertheless, its relatively high conservation, as in the case of helix A, may be explained by the significant role of helices in maintaining the correct structure of p17. It is known that due to the presence of specific structural motifs defined as “coiled-coil” sequences, p17 exhibits a high propensity for misfolding and aggregation [18]. This HIV-1 matrix protein is known to possess self-interaction properties, as it forms trimers and hexamers [19,20,21]. The main identified sites for aggregation are the C-terminal region (capable of interacting with the same region of other p17 molecules [22]) and the central part of the matrix (amino acids 42–47) [23], which partially coincides with the identified conserved regions CR_3–4. It is likely that the high conservation of these regions is necessary to prevent excessive aggregation of p17 molecules and subsequent disruptions in capsid assembly.

**Capsid Protein**

The HIV capsid protein is represented within the Pr55 polyprotein by the CA domain, which in our alignment corresponds to coordinates 144–374 (HXB2: 132–362) and, upon final maturation, forms the p24 protein. The CA domain partially includes region CR_5 and entirely encompasses 12 identified conserved regions, CR_6–17. Among these, five are located in the N-terminal capsid domain (CA-NTD, HXB2: 142–249), one is partially within the linker region (HXB2: 250–274) and partially within the C-terminal capsid domain (CA-CTD, HXB2: 275–348), and five are entirely within the CA-CTD. Interactions between CA domains within Gag play a central role in forming the immature lattice observed in the budding particle. The assembly of CA proteins into a fullerene structure, consisting of approximately 250 CA hexamers and 12 CA pentamers, forms the viral capsid. It serves as a protective shell for the viral genomic RNA inside the mature particle and during entry into the cell cytoplasm [24,25].

The CA-NTD consists of seven α-helices and a cyclophilin A (CypA) binding loop, with an N-terminus that is unstructured in Gag, but folds into a β-hairpin in mature CA [26,27]. The CA-NTD forms a stable inner core of the hexamer, with the β-hairpin essentially lining the interior of its center, the R18 pore. This pore is a size-selective, positively charged channel that facilitates the diffusion of nucleotides into the capsid core while simultaneously preventing access to nucleases, host cell restriction factors, and sensors [28].

The CA-CTD consists of a helix, the major homology region (MHR) loop, which is highly conserved and essential for viral replication, and four α-helices. The C-terminus of the CA-CTD undergoes an important structural rearrangement during viral maturation [29,30]. The C-terminal domains form a flexible outer ring of hexamers, playing a crucial role in forming interfaces for hexamer assembly and for contacts with other hexamers [31].

In addition to the R18 pore, there is a critical interprotomer pocket that forms between hexameric subunits and is generated by the interaction of the NTD of one subunit with the CTD of another. This NTD-CTD interprotomer interface is present in the mature HIV-1 capsid and is crucial for proper capsid assembly, stability, and recognition of host cell factors [32]. Evidence suggests that this pocket binds host cell factors involved in nuclear import, indicating that intact CA oligomers are imported into the nucleus during nuclear entry of the preintegration complex (PIC) [33]. Amino acid residues comprising CR_17, which captures the end of the CA domain within Gag (specifically CTD helix H10), play an important role in interface formation. Additionally, CTD helix H9, which coincides with CR_16, plays a significant role in forming CTD-CTD interfaces within the hexamer [34].

Additionally, CR_15–17 may participate in interactions with human lysyl-tRNA synthetase for the selective packaging of tRNA-Lys, which plays a key role in initiating HIV reverse transcription. For the remaining identified conserved regions, no precise correspondence to described functional domains could be established. However, based on the observed relationships, it can be inferred that they are also critical for the functioning of the R18 pore (CR_5–6) and for the formation of NTD-CTD interfaces (CR_7–15) [35].

**Nucleocapsid Protein**

The Gag nucleocapsid domain (align. coordinates 390–445, HXB2: 376–430) and its corresponding mature protein form (NCp7) are basic polypeptides of 55 amino acids. They are characterized by the presence of two conserved structures, zinc fingers (ZF), containing the invariant CCHC motif. The ZFs are separated by a short linker and flanked by small domains rich in basic residues.

Among the identified conserved regions, four are entirely within NC (CR_19–22), and one is partially included (CR_23). Specifically, CR_19 corresponds to the first zinc finger, including the amino acid preceding it. CR_20 encompasses the end of the zinc finger and part of the linker sequence. CR_21–22 are entirely within the second zinc finger, including the amino acid preceding it. Finally, CR_23 comprises the last six amino acids of the NC domain [36]. The zinc fingers and the hydrophobic plateau formed upon ZF folding bind to the backbone and nitrogenous bases of nucleic acids. Moreover, the high flexibility of the protein enables it to bind to a wide variety of nucleic acid sequences [37,38,39,40]. The importance of preserving NC structure explains the high sequence conservation across HIV-1 subtypes and isolates from treated patients, as well as the low probability of detecting mutations [41,42].

**Gag p6**

Gag p6 is a multifunctional domain that plays a key role in the late phase of the viral cycle. It typically comprises approximately 52 amino acids located at the C-terminus of the Gag polyprotein, corresponding to alignment coordinates 464–543 (HXB2: 447–498). This domain contains the identified conserved region CR_25, situated at the C-terminal end of the domain. This conserved region participates in Env binding and regulates the packaging of cleaved Pol proteins [43,44,45]. Functional domains are also known to exist at the N-terminus of p6. However, their function is primarily associated with amino acid motifs rather than strictly conserved sequences [16,46,47,48]. The role of the central region of p6 remains unclear and is likely non-essential for p6 function insofar as its polymorphism does not impair either viral infectivity or replication kinetics [49].

**Spacer Regions SP1 and SP**

The Gag spacer regions SP1 (align. coordinates 375–389, HXB2: 363–375) and SP2 (align. coordinates 446–463, HXB2: 431–446) are regulators of Gag assembly and maturation. Within SP1 (a conserved region), CR_18 was identified, which is presumably associated with the functional role of this domain. Mutations in the first seven residues of SP1 proximal to the CA domain are known to disrupt viral assembly and lead to the formation of tubular structures containing unprocessed Gag at the plasma membrane [50,51]. A single conserved region, CR_24, was also identified within the SP2 region. Although the role of this region has not been fully elucidated, the identified conserved region is most likely associated with Gag processing and proper targeting of the HIV protease.

#### 3.2.2. Polymerase

Whereas the structural proteins of HIV-1 are initially translated as part of the Gag polyprotein, the enzymes that enable the virus to replicate and spread are synthesized as part of the Gag-Pol precursor polyproteins. Both polyproteins are cleaved by the viral protease (PR), which is itself synthesized as part of Gag-Pol during maturation [52]. Pol is processed to yield the viral enzymes PR, reverse transcriptase (RT), and integrase (IN). Gag-Pol is synthesized via translational readthrough; different retroviruses employ various readthrough mechanisms. HIV-1 utilizes a ribosomal frameshifting mechanism. The efficiency of this frameshift results in a Gag:Gag-Pol ratio of approximately 20:1 [53,54,55,56], making Gag-Pol a relatively minor component of the virion (~100 copies per virion) [57]. Because the enzymes comprising Pol are key participants in the viral life cycle, they are also targets for antiretroviral therapy. As the Pol polyprotein is the precursor to these viral enzymes, it represents an extremely conserved region, and the conducted analysis enabled the identification of 49 highly conserved regions associated with the functional domains of the mature enzymes (Table 7). The coordinates used correspond to alignments of the full polyprotein rather than its individual cleavage products; the same applies to the HXB2 reference sequence coordinates. However, the subsequent description of conserved regions will be structured according to the Pol cleavage products.

**Retroviral Aspartyl Protease**

HIV-1 protease is a member of the aspartic protease family due to the presence of the conserved catalytic Asp-Thr/Ser-Gly triad [58]. The mature protease is catalytically active as a dimer composed of two 99-residue subunits, with each subunit contributing one copy of the catalytic triplet. PR recognizes specific amino acid sequences at various cleavage sites within the Gag and Gag-Pol polyproteins and hydrolyzes the peptide bonds to release the individual structural proteins and enzymes. These cleavage sites must be hydrolyzed in the correct sequential order to generate an infectious virus [59,60,61].

The identified Pol_CR_1 contains the beginning of the Pol domain sequence corresponding to the PR, including the 4 amino acids preceding it and the first 5 residues of the enzyme. The subsequent conserved regions Pol_CR_2–5 are located within PR. Pol_CR_1 and Pol_CR_5 are part of the interface between monomers of the protease dimer, which upon folding is formed by the N- and C-termini of the monomer (alignment coordinates: 86–90 and 181–184). Pol_CR_2 is associated with hinge points in the fulcrum element of the enzyme and also encompasses the conserved catalytic DTG triplet. Pol_CR_3 corresponds to the active site flaps, which modulate substrate entry into the active site cavity and consist of two β-hairpin loops (alignment coordinates: 128–141). Pol_CR_4 comprises the sequence lining the interior of the enzyme active site, situated between the cantilever and an α-helix [62].

According to genotype and phenotype data available in HIVdb [63,64], the major protease inhibitor resistance mutations are located within the Pol_CR_3 region. This aligns with the mechanism of action of these inhibitors: they typically enter the active site and block it, whereas altered active site flaps prevent this access, leaving the enzyme active site free. However, these same changes negatively impact enzyme activity. Consequently, such mutations are maintained by selection only under the pressure of antiretroviral drugs.

**Reverse Transcriptase**

The RT enzyme is represented in Pol by several domains: the retroviral reverse transcriptase (RT_Rtv), the RT thumb domain (RVT_thumb), the reverse transcriptase connection domain (RVT_connect), and the retroviral ribonuclease H (RNase_H). The RT_Rtv, RVT_thumb, and RVT_connect domains together constitute the polymerase proper and contain the fingers, palm, thumb, and connection motifs of reverse transcriptase [65,66,67]. The RNase H domain facilitates degradation of RNA from the DNA-RNA duplex during reverse transcription. Other functions of RNase H include the removal of tRNA^3Lys^ and the removal of the polypurine tract (PPT), which serve as primers for negative-strand DNA synthesis and positive-strand DNA synthesis, respectively [68,69,70].

Conserved regions Pol_CR_6 through Pol_CR_31 are located within reverse transcriptase, with Pol_CR_6–15 corresponding to the RT_Rtv domain; Pol_CR_16–18 to the RVT_thumb domain; Pol_CR_20–24 to the RVT_connect domain; and Pol_CR_25–31 to the RNase_H domain. Three major studies [71,72,73] provide detailed reviews of conserved patterns in RT. The conserved regions identified in those studies generally coincide with those identified in our current analysis. However, some discrepancies exist, mainly consisting of certain large regions in our study being subdivided into several smaller ones due to separation by less conserved areas. Since the nature of conservation in these regions has been thoroughly described in those works, we will not dwell in detail on the coinciding regions and will instead focus only on those that differ.

Regions Pol_CR_7–9 are located within the RT_Rtv domain and were not annotated as conserved in a study [71]. Nevertheless, the amino acids comprising these regions were noted as highly conserved but, for some reason, were not delineated by the authors as distinct conserved areas. In a different study [74], however, these regions are identified as conserved regions of RT. All three conserved regions are part of DNA-binding elements and, evidently, play a key role in this process, which accounts for their high conservation.

**Integrase**

Integrase (IN) is a crucial viral enzyme consisting of 288 amino acids and encoded by the 3′-end of the HIV polymerase gene. Integrase catalyzes the integration of newly synthesized double-stranded DNA into the host genomic DNA. It also plays a role in stabilizing the preintegration complex (PIC), which consists of the 3′-processed viral genome and one or more cellular cofactors involved in PIC nuclear import [75]. HIV IN comprises three main domains: the integrase zinc binding domain (IN_Zn); the integrase core domain (retroviral-like integrase, rve); and the integrase DNA binding domain (IN_DBD_C). The rve domain also contains the transposase InsO and inactivated derivatives (Tra5) region, which exhibits transposase activity.

A total of 18 conserved regions were identified within IN: Pol_CR_32–49. Regions Pol_CR_33 and Pol_CR_34 belong to the IN_Zn domain and are part of the zinc fingers, indicating conservation not only of positions corresponding to the IN_Zn motif, but also of the overall zinc finger structure. Regions Pol_CR_35–45 correspond to the integrase core domain, with Pol_CR_39–45 residing within the region possessing transposase activity. A study [76] described several of the identified conserved regions within the core domain and DBD_C domain, specifically: Pol_CR_36, 37, 40, 41, 47, and 48. However, the remaining conserved regions also play important roles in IN function. For instance, region Pol_CR_36 performs a critical structural role, linking the core domain and the C-terminal DBD domain within helix α6. Regions Pol_CR_42–45 are part of β-sheets 1–5, which are essential for proper folding of the core domain and for correct communication between the catalytic core and the DNA-binding domain.

#### 3.2.3. Envelope

HIV-1 Env (gp160) consists of two subunits, gp120 and gp41, along with a spacer peptide that is cleaved off during proteolysis. The gp120/gp41 complex is presented on the virion surface as trimers. Env directs the fusion of viral and cellular membranes to initiate infection of a susceptible cell [77]. Conformational changes accompany the binding of the native Env trimer to the receptor (CD4) and coreceptor (e.g., CCR5 or CXCR4), leading to a cascade of refolding events in gp41. Subsequent folding of the C-terminal region of gp41 into a hairpin conformation creates a post-fusion six-helix bundle [78,79], which brings the viral and cellular membranes together, resulting in fusion and viral entry.

**gp120**

Of the twenty-eight highly conserved regions identified within the Env polyprotein (Table 10), sixteen (Env_CR_1–16) correspond to the gp120 subunit. This protein is well known for the high variability of its epitopes; nevertheless, regions exhibiting conservation are also quite distinctly delineated [80]. Among these, the first three are located in the N-terminal region of the protein and correspond primarily to hydrophobic areas of gp120 with a not yet fully understood functional role. Env_CR_4 coincides with the β1 strand, preceding the α1 helix which contains the conserved region Env_CR_5. The conserved region Env_CR_6 is situated in the area corresponding to the V1/V2 loop and represents a region of high conservation within this relatively variable loop. The next conserved region (Env_CR_7) belongs to the β3 strand, located immediately after the V1/V2 loop. Conserved regions Env_CR_8–9 are found in the β4 and β5 strands, respectively. Following these strands lies the variable loop A region. Conserved region Env_CR_10 corresponds to the β7 and β8 strands. Region Env_CR_11 encompasses the B loop and the β9 strand. Further on, between regions Env_CR_11 and Env_CR_12, there is an extended variable region, and the twelfth conserved region includes the β16 and β17 strands. Subsequently, region Env_CR_13 corresponds to the β19 and β20 strands, region Env_CR_14 to the β21 strand, and Env_CR_15 pertains to the final helix, α5. Env_CR_15 corresponds to a hydrophilic region at the end of gp120, which constitutes the cleavage site between gp120 and gp41.

A similar structure of conserved and variable regions has been described [81]. Clearly, the conserved areas of gp120 are, first and foremost, important structural elements required for the formation of correct monomers and subsequently for gp120 trimers. Partially conserved regions are also found in the interfaces involved in host receptor binding. However, it is also known that key areas within the structure of such interfaces are considerably variable.

**gp41**

The remaining conserved regions (Env_CR_17–28) correspond to gp41 sequence. Within these, Env_CR_17–22 belong to the N-terminal part of the protein, encompassing regions responsible for membrane fusion. The subsequent regions correspond to the C-terminus, which includes the transmembrane and intracellular domains of gp41. The conserved region Env_CR_17 is located within the N-terminal fusion peptide, which is responsible for merging the viral membrane with the host membrane. The following regions, Env_CR_18–22, are part of the extended heptad repeat region 1, which precedes the cysteine loop region containing the conserved regions Env_CR_23 and Env_CR_24. Subsequently, after a long variable region, Env_CR_25 begins, corresponding to the membrane-proximal external region. The conserved region Env_CR_26 is situated within the transmembrane region, and regions Env_CR_27–28 are located within the cytoplasmic domain. A similar conservation pattern for gp41 has been reported [82]. The authors emphasize the high conservation of the gp41 ectodomains and associate this with the high functional relevance of this region.

#### 3.2.4. Viral Infectivity Factor

Vif is a small, intrinsically disordered protein with a molecular mass of approximately 23 kDa, consisting of roughly 192–216 amino acids depending on HIV-1 subtype. Vif acts as a substrate receptor for A3 proteins within the Cullin–RING E3 ubiquitin ligase complex. E3 Cullin–RING complexes represent the largest subfamily of RING domain-containing ligases. These complexes target cellular proteins for ubiquitin-mediated degradation via the 26S proteasome [83]. Two functionally distinct domains of Vif mediate its role as a structural hub for the complex, the N-terminal A3 substrate-binding domain (referred to as the α/β domain) and the C-terminal adapter domain (α domain), which interacts with the E3 ligase machinery. The Vif α/β domain forms a central five-stranded antiparallel β-sheet (strands β2–β6), flanked on its convex side by three helices (α1, α2, α5). The α domain contains two α-helices and includes a BC-box motif, as well as the conserved HCCH motif. The HCCH motif contains His and Cys residues that tetrahedrally coordinate a single Zn^2+^ ion with high affinity [84,85,86].

Analysis of the Vif sequence alignments enabled the identification of six highly conserved regions, which are presented in Table 13. The first conserved region, located at HXB2 coordinates 1–16, resides in the N-terminal domain of the protein and corresponds to one of the A3F protein binding sites. Vif_CR_6 also participates in forming this site upon Vif folding. The A3G protein binding site is primarily formed by Vif_CR_2 and Vif_CR_5; however, the region corresponding to HXB2 coordinates 40–45 is also known to participate in this interaction. Nevertheless, this region was not classified as conserved in our analysis, as its conservation was observed only within individual subtypes and diminishes significantly when comparing sequences from different subtypes. Additionally, Vif_CR_3 contributes to the formation of the A3 protein-binding domain. The fourth conserved region constitutes the Elongin C binding site, a component of the E3 ubiquitin ligase complex.

Beyond the aforementioned HXB2 40–45 region, the Vif protein contains numerous other areas that exhibit high conservation within subtypes but display considerable inter-subtype variability. This may indicate active adaptation of Vif during viral evolution and could contribute to differential infectivity among HIV-1 subtypes.

#### 3.2.5. Viral Protein R

The product of the accessory gene *vpr* encodes a 14 kDa protein consisting of 96 amino acid residues; it is expressed during the late stages of viral replication from an open reading frame located in the central region of the viral genome. This protein is highly conserved among primate lentiviruses, including HIV-1, HIV-2, and simian immunodeficiency virus, supporting the hypothesis that it plays an important role in the viral life cycle [87]. Although our understanding of how Vpr contributes to HIV-1 replication and pathogenesis remains incomplete, one well-characterized mechanism involves the recruitment of the CRL4 E3 ubiquitin ligase and its substrate receptor DCAF1 (CRL4 DCAF1) to deplete cellular proteins that directly or indirectly target viral components [88]. Nevertheless, other potential functions of Vpr have been proposed, including facilitation of nuclear pore transport, participation in preintegration complex formation, and induction of cell cycle arrest at the G2 phase, among others.

The functional domains of the protein remain poorly characterized. Therefore, it is not possible to definitively associate the identified conserved regions with specific Vpr functions. However, it can be noted that two regions, Vpr_CR_2 and Vpr_CR_3, are located within the hydrophobic core, which consists of several sterically converging hydrophobic residues from helices α1 and α3. Both regions are situated at the boundary between organized helices and disordered regions and are likely important for stabilizing the complex structure. Vpr_CR_1 and Vpr_CR_4 are located in disordered regions at the N- and C-termini of Vpr, respectively, yet their functional roles remain unclear.

#### 3.2.6. Regulator of Virion Expression

Rev is a protein consisting of 116 amino acids (~13 kDa) on average. It is encoded by two exons that overlap with other genes: *tat* and *env* [89,90]. Rev plays a crucial role in the viral life cycle by coordinating the nuclear export of unspliced and incompletely spliced viral mRNAs [91]. Rev, expressed from fully spliced viral RNA, translocates to the nucleus, binds to the Rev response element (RRE) through oligomerization, and induces the export of incompletely spliced mRNAs to the cytoplasm [89,91,92]. Rev is also involved in RNA splicing, stability, and translation. However, its impact on these processes remains poorly understood [91].

The four most conserved regions we identified (Table 26) are located within the N-terminal portion of Rev and are situated in the N-terminal domain itself (Rev_CR_1), as well as within the oligomerization domain, the turn region (Rev_CR_2–3), and the arginine-rich motif (ARM) domain (Rev_CR_4). The ARM domain represents the most highly conserved region of the Rev protein [93]. This finding is consistent with the results of competitive deep mutational scanning, in which the majority of residues within the ARM domain exhibited strong selection [90]. Therefore, it can be inferred that the well-conserved structure of the ARM domain is essential for Rev function. The N-terminal region as a whole also demonstrated a high degree of conservation, which may be explained by its overlap with the functionally significant arginine-rich motif of the Tat protein [90,94].

#### 3.2.7. Trans-Activator of Transcription

Tat is a short protein (averaging 101 residues) expressed during the early stages of infection, initially described as a transactivator of HIV-1 genes [95]. However, another intriguing property of Tat is its extracellular role and high level of secretion from HIV-1-infected cells, suggesting that it plays an important part in HIV-1′s ability to evade the immune response in infected patients [96]. Extracellular Tat penetrates the membrane of numerous uninfected bystander cells, such as cytotoxic T lymphocytes, to induce apoptosis [97].

Six major regions are distinguished within the Tat structure [98]. The conserved regions we identified (Table 27) correspond to region I, located at the N-terminus of the protein (Tat_CR_1), as well as to regions III and, predominantly, IV (Tat_CR_2). Other studies have highlighted the high functional significance of region IV. It is required for crossing the cell membrane and binding to the trans-activating response element, a stem-loop structure at the 5′-end of viral mRNA. The role of region I has not been fully elucidated. However, based on its involvement in folding the central β-sheet of the protein, this region primarily serves a structural function for Tat.

#### 3.2.8. Negative Regulatory Factor

Nef is a myristoylated peripheral membrane protein of 23–35 kDa (~206 amino acid residues in most HIV-1 strains) expressed by primate lentiviruses. The full-length structure of Nef consists of six α-helices (α1–α6) and a β-sheet composed of five antiparallel β-strands (β1–β5). Nef structure is divided into four units: a flexible myristoylated membrane-anchoring region of variable length (residues 1–56); followed by the PxxP loop (residues 57–80); the core domain (residues 81–206, with Δ148–180); and a flexible C-terminal loop (residues 148–180). While the anchor and the flexible C-terminal loop are not conformationally constrained relative to the core domain, the PxxP loop is weakly associated with the core domain through hydrophobic interactions [99].

The identified region Nef_CR_1 is located at the very beginning of the N-domain responsible for anchoring Nef to the membrane. This highly conserved region and its myristoylation are known to be critically important for membrane localization of the protein. Within the proline-rich loop of the N-terminus, two conserved regions, Nef_CR_2–3, can be observed at the beginning and end of the loop. The proline-rich motif itself mediates interactions between Nef and signaling molecules, such as Hck and Vav, and is central to Nef’s ability to induce cellular activation, a function that may be necessary for maintaining viral replication in resting cells [100,101]. However, the identified conserved regions may also be required for protease cleavage at the loop termini during final Nef maturation [102]. The next highly conserved regions identified, Nef_CR_4 and Nef_CR_5, are part of the Nef core domain and are primarily associated with the SH3 binding site. The latter is necessary for interactions with Fyn and MHC class I molecules, and this interaction is apparently important for Nef function [103] Nef_CR_6 belongs to the C-terminal loop of the protein, which likely also participates in interactions with AP-1 and AP-2 molecules to influence MHC class I. However, the function of this loop is insufficiently characterized to definitively assess the role of the identified conserved region in its activity.

#### 3.2.9. Viral Protein U

The HIV-1-specific Vpu protein is an integral class I membrane phosphoprotein consisting of 81 amino acids. Its amino acid sequence exhibits extensive diversity, which often increases as infection progresses. Key conserved functions of Vpu include downregulation of CD4, antagonism of tetherin (BST-2/CD317), and modulation of other cellular receptors [104,105,106]. Vpu is amphipathic in nature and consists of a hydrophobic N-terminal membrane anchor located adjacent to a polar C-terminal cytoplasmic domain.

Despite the presence of conserved intra-subtype regions within the transmembrane domain of Vpu, the areas exhibiting the highest conservation across all subtypes are located within the cytoplasmic domain of the protein. The highly conserved region identified during analysis is situated entirely within the second α-helix of the cytoplasmic domain. A distinctive feature of this region is a dileucine-like sorting motif. This motif functions as an intracellular trafficking signal. It influences protein localization and is required for efficient removal of tetherin from the cell surface, as well as for controlling the volume of virus-containing compartments in macrophages [104,105,106,107,108]. Mutations at positions G59 and E62 also impair the ability of Vpu to suppress tetherin-induced NF-κB signaling [104].

### 3.3. Study Limitations

The authors recognize that the present study has several limitations that should be considered when interpreting the results and planning future investigations. In this work, the analysis was focused on sequences belonging to HIV-1 Group M, which predominates in the global epidemiology of HIV infection. Groups O, N, and P were not included due to their extremely limited representation in public databases in the form of full-length genomes with sufficiently reliable annotation quality.

In addition, within Group M, the number of sequences available for different subtypes varied substantially. For subtypes A2, F2, J, and K, fewer than 15 full-length genomes met the inclusion criteria, which may have affected the statistical robustness of the analyses. Therefore, results obtained for low-representation subtypes should be interpreted with particular caution, and conclusions regarding pan-subtype conservation require validation using expanded datasets.

The present study attempted to identify conserved regions using a unified statistical threshold applied across all proteins regardless of differences in the biological and evolutionary pressures acting on them. Although many of the identified conserved regions corresponded to functionally important domains previously described in the literature, this approach may underestimate conservation in certain protein regions. Consequently, the results should be interpreted in conjunction with complementary studies addressing the structural, biochemical, and physicochemical properties of HIV-1 proteins. Similarly, the minimum conserved-region length threshold (5 amino acids) was selected empirically to prioritize extended conserved regions; therefore, shorter biologically significant motifs may have been overlooked.

Finally, this study is purely bioinformatic in nature and should be regarded as a framework for prioritization of hypothetical targets rather than direct translational validation. The identified conserved regions do not by themselves guarantee suitability for the development of diagnostic primers, vaccine immunogens, or therapeutic antibodies. Additional experimental studies are required to evaluate their accessibility, immunogenicity, and functional relevance in appropriate biological systems.

## 4. Materials and Methods

### 4.1. Materials

**Figure 20:**
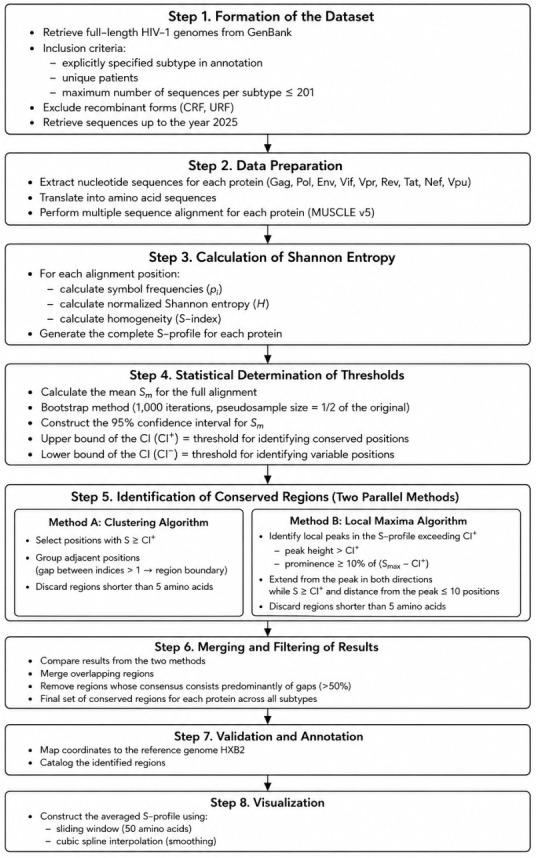
Workflow of the computational pipeline used for identification of conserved regions in HIV-1 proteins.

We downloaded random full-length HIV-1 genomic sequences from the NCBI Nucleotide database, selected according to several criteria: sequences were deposited up to the year 2025; HIV strains were obtained from different patients; and the annotations explicitly indicated viral subtype and the boundaries of genomic regions. In this study, we focused on sequences belonging to subtypes of group M, as this group is the most prevalent in the population; therefore, belonging to other groups was considered an exclusion criterion. Additionally, the number of sequences per viral subtype was limited to a maximum of 201. A total of 1119 sequences were included in the study (Table 28). An overview of the next steps in the analysis is provided in Figure 20.

### 4.2. Normalized Shannon Entropy

Prior to analysis, multiple sequence alignment of the investigated sequences was performed using the MUSCLE v5 algorithm [109]. Subsequently, amino acid frequencies were calculated for each column of the multiple sequence alignment, based on which the Shannon entropy (1) was computed. For amino acid sequences, all symbols represented in GenBank translations, as well as the gap symbol, were taken into account.

(1) $$H=-\sum_{i=1}^{q}p_{i}log_{2}\;p_{i}$$

H—Shannon index;
q—maximum possible number of distinct symbols;
$p_{i}$—frequency of a given symbol in an alignment column.

Additionally, normalization was performed against the maximum possible entropy. The normalized entropy measure was calculated as follows (2).

(2) $$H(norm)=\sum_{1}^{q}p_{i}log_{q}\;p_{i}$$

H(norm)—normalized Shannon index;
q—maximum possible number of distinct symbols;
$p_{i}$—frequency of a given symbol in an alignment column.

For the analysis, the amino acid residue homogeneity measure S was used (3).

(3) S=1−H(norm)

S—amino acid residue homogeneity;
H(norm)—normalized Shannon index.

The next stage involved analysis of the resulting amino acid residue heterogeneity profile. For this purpose, the profile of S-values obtained for each position (the full S-profile) was used.

### 4.3. Ranking and Threshold Methods

The complete S-profile can be represented as a histogram (Figure 1), wherein each alignment column is assigned an S-index value. It is evident that the more heterogeneous the investigated alignment, the smaller the area of the figure bounded by the upper edge of the histogram bars. Since the area of each individual bar equals the S-value at that position, the total area corresponds to the sum of S-values across all alignment columns. Normalizing this value by the maximum possible area, which would equal one, yields the S_m_ value for the entire alignment. Based on this reasoning, we selected it as a metric reflecting the overall sequence similarity within the alignment.

Furthermore, it was hypothesized that S_m_ represents a meaningful measure of conservation for an alignment fragment or the entire alignment. To more distinctly identify highly conserved and highly variable positions, a confidence interval at a 95% significance level was determined for S_m_, with its upper and lower bounds serving as the primary cutoff thresholds for detecting regions exhibiting differential conservation. For amino acid sequences, the threshold corresponding to the upper bound of the confidence interval determined collectively for all protein products was applied.

### 4.4. Conserved Region Detection

Two independent algorithms based on analysis of the S-index profile were used to identify conserved regions within the sequences. The Clustering Algorithm was applied as the primary method to identify extended conserved domains by grouping adjacent positions. All positions with an S-index value exceeding the upper bound of the 95% confidence interval were considered candidate conserved sites. Their indices in the ordered sequence were used to determine breaks as follows. Positions were separated into distinct regions where the gap between indices exceeded 1, allowing the delineation of natural conservation clusters while ignoring isolated non-conserved insertions. To eliminate random short clusters, only regions with a minimum length of 5 amino acid positions were retained for further analysis. This approach is effective for detecting extended conserved blocks with well-defined boundaries.

**The Peaks Algorithm** was selected as a complementary method, aimed at identifying local conservation maxima. Local maxima were identified in the S-index profile according to the following criteria: the peak height had to exceed the upper bound of the 95% confidence interval; and its relative prominence had to be at least 10% of the difference between the maximum S-index value and the threshold, ensuring statistical significance. The minimum distance between peaks was set to 10 positions. From each detected peak point, expansion was performed in both directions along the sequence as long as S-index values remained above the threshold and the distance from the peak did not exceed 10 positions, preventing excessive merging of adjacent peaks. As with the first method, the final regions were filtered by a minimum length of 5 positions. This approach enables the identification of compact, yet highly conserved, motifs that might be overlooked when analyzing average values over extended regions.

Conservative regions confirmed by both algorithms that included regions consisting predominantly of gaps (i.e., insertions occurring in a small number of sequences) were filtered out from the overall list.

### 4.5. Statistics

To assess the distribution of S_m_ within a selected alignment region, the bootstrap method was employed with a pseudosample size equal to half of the original dataset. To ensure accuracy in estimating the distribution, the number of bootstrap iterations was set to 1000. The 95% confidence interval of the distribution was selected as the confidence interval for S_m_. This approach provided a statistically justified basis for determining the threshold values subsequently used to identify conserved and variable regions within the alignments.

### 4.6. Visualizations

For visualization of the obtained results, the averaged S-profile was used as it is more convenient for visual assessment of the data and is associated with the value over a sequence interval. Two methods were employed to display the averaged profile: a moving average heatmap and a line plot generated using cubic spline interpolation. This method allows approximation of the original data with a high degree of smoothness, particularly in regions where the S-index varies unevenly. Cubic spline interpolation is especially useful for noise reduction and identifying long-term trends in the data.

The use of splines is justified in bioinformatics research, where data are dense and preserving both global and local trends is crucial for interpretation. This method is recommended as a powerful tool for the analysis and visualization of genetic sequences due to its flexibility and mathematical precision. Images were generated using the Python v3.12 and libraries Plotly v3.0.1 and Matplotlib v 3.10.

## 5. Conclusions

In the present study, an algorithm for screening and analysis of amino acid sequence conservation was developed and validated. This approach is based on calculation of the normalized Shannon index, followed by the application of two complementary methods: clustering and local maxima detection. The employed approach enabled not only the quantitative assessment of sequence homogeneity, but also the identification of localized regions exhibiting extreme conservation values, including compact motifs that might have been overlooked when using traditional sliding window analytical methods. The application of the bootstrap method for confidence interval calculation provided statistical justification for threshold values. The use of cubic spline interpolation for visualization effectively reduced noise impact and revealed long-term trends in the distribution of conserved regions.

Application of the algorithm to HIV-1 protein alignments revealed a wide range of homogeneity index values: from 0.784 for the highly variable Vpu protein to 0.920 for the highly conserved Pol polyprotein. This reflects differences in functional constraints, evolutionary age of genes, and the intensity of selective pressure from the immune system and antiretroviral therapy. The highest conservation was observed for enzymatic and structural proteins (Pol, Gag, Vpr). Regulatory proteins (Rev, Tat) and proteins interacting with the immune system (Env, Vpu) demonstrated significantly higher variability, with conserved regions clearly localized within functionally significant domains.

As a result of the analysis, the major conserved regions were cataloged for all investigated proteins. The identified regions in most cases correspond to known functional domains such as enzyme catalytic centers, zinc fingers, sites of interaction with cellular factors, membrane-binding regions, and signaling motifs. This correspondence confirms the validity of the employed approach. Regions conserved across all subtypes are of particular value insofar as they represent promising targets for the development of therapeutic strategies and diagnostic systems effective in the context of global HIV-1 diversity. The obtained results can be used to optimize existing approaches and develop new strategies for HIV therapy and diagnostics.

## Figures and Tables

**Table 1:** Statistical analysis of S-index values for establishing cutoff thresholds for subsequent analysis.

Subtype	S_m_	CI−	CI+
All sequences	0.8746	0.8671	0.8822
A1	0.9158	0.9098	0.9227
A2	0.9030	0.8961	0.9095
A6	0.9270	0.9208	0.9327
B	0.9312	0.9257	0.9370
C	0.9086	0.9017	0.9153
D	0.8812	0.8733	0.8890
F1	0.9063	0.8989	0.9133
F2	0.9008	0.8933	0.9075
G	0.8685	0.8602	0.8757
H	0.9230	0.9163	0.9289
J	0.8717	0.8642	0.8792
K	0.8356	0.8287	0.8427

**Table 2:** S-index statistics for Gag protein aligns.

Subtype	S_m_	CI−	CI+
All sequences	0.9058	0.8896	0.9209
A1	0.9393	0.9269	0.9516
A2	0.9248	0.9105	0.9383
A6	0.9459	0.9348	0.9560
B	0.9517	0.9423	0.9605
C	0.9242	0.9087	0.9374
D	0.9087	0.8937	0.9223
F1	0.9216	0.9074	0.9347
F2	0.9216	0.9064	0.9368
G	0.9143	0.8991	0.9300
H	0.9432	0.9309	0.9547
J	0.9087	0.8951	0.9228
K	0.9270	0.9128	0.9404

**Table 3:** Statistics of conserved regions detected in Gag.

Subtype	Conserved Regions	Mean Conserved Region Length	S_m_ in Conserved Regions
All sequences	26	8.35	0.9856
A1	24	10.67	0.9953
A2	27	7.67	1.0000
A6	24	9.08	0.9957
B	30	8.13	0.9949
C	26	8.12	0.9952
D	25	7.92	0.9863
F1	25	9.24	0.9912
F2	23	8.52	1.0000
G	28	8.71	0.9896
H	24	10.04	0.9982
J	25	8.04	0.9909
K	23	7.96	1.0000

**Table 4:** Conserved region summary for Gag polyprotein aligns.

Region Name	HXB2 Coordinates	Alignment Coordinates	Length	S_m_
Gag_CR_1	1–6	1–6	6	0.9979
Gag_CR_2	21–25	21–25	5	0.9916
Gag_CR_3	36–45	36–45	10	0.9871
Gag_CR_4	96–101	96–101	6	0.9812
Gag_CR_5	128–136	140–148	9	0.9708
Gag_CR_6	147–157	159–169	11	0.9900
Gag_CR_7	176–180	188–192	5	0.9777
Gag_CR_8	190–201	202–213	12	0.9878
Gag_CR_9	203–213	215–225	11	0.9830
Gag_CR_10	230–240	242–252	11	0.9959
Gag_CR_11	260–278	272–290	19	0.9841
Gag_CR_12	280–284	292–296	5	0.9858
Gag_CR_13	286–299	298–311	14	0.9972
Gag_CR_14	303–308	315–320	6	0.9880
Gag_CR_15	319–324	331–336	6	0.9843
Gag_CR_16	326–330	338–342	5	0.9924
Gag_CR_17	342–355	354–367	14	0.9938
Gag_CR_18	362–367	374–379	6	0.9928
Gag_CR_19	389–398	403–412	10	0.9876
Gag_CR_20	402–407	416–421	6	0.9800
Gag_CR_21	410–415	424–429	6	0.9967
Gag_CR_22	417–424	431–438	8	0.9885
Gag_CR_23	425–433	440–448	9	0.9741
Gag_CR_24	440–446	457–463	7	0.9836
Gag_CR_25	487–495	532–540	9	0.9821

**Table 5:** S-index statistics for Pol protein aligns.

Subtype	S_m_	CI−	CI+
All sequences	0.9200	0.9102	0.9286
A1	0.9592	0.9523	0.9661
A2	0.9463	0.9376	0.9547
A6	0.9617	0.9544	0.9683
B	0.9654	0.9593	0.9711
C	0.9544	0.9466	0.9613
D	0.9360	0.9271	0.9444
F1	0.9462	0.9377	0.9546
F2	0.9444	0.9357	0.9532
G	0.8812	0.8732	0.8887
H	0.9565	0.9491	0.9635
J	0.9357	0.9265	0.9452
K	0.7588	0.7517	0.7661

**Table 6:** Statistics of conserved regions detected in Pol.

Subtype	Conserved Regions	Mean Conserved Region Length	S_m_ in Conserved Regions
All sequences	50	9.96	0.9709
A1	50	11.74	0.9971
A2	54	10.15	1.0000
A6	53	10.21	0.9963
B	49	11.14	0.9965
C	46	11.17	0.9965
D	47	9.89	0.9890
F1	51	9.98	0.9945
F2	48	9.85	1.0000
G	48	10.46	0.9278
H	50	10.52	0.9961
J	44	8.18	1.0000
K	30	8.60	0.8091

**Table 7:** Conserved region summary for Pol polyprotein aligns.

Region Name	HXB2 Coordinates	Alignment Coordinates	Length	S_m_
Pol_CR_1		82–90	9	0.9703
Pol_CR_2		102–115	14	0.9696
Pol_CR_3	11–21	127–137	11	0.9713
Pol_CR_4	48–53	164–169	6	0.9723
Pol_CR_5	59–67	175–183	9	0.9730
Pol_CR_6	78–83	194–199	6	0.9728
Pol_CR_7	85–95	201–211	11	0.9656
Pol_CR_8	114–123	230–239	10	0.9710
Pol_CR_9	127–131	243–247	5	0.9645
Pol_CR_10	134–165	250–281	32	0.9684
Pol_CR_11	168–184	284–300	17	0.9684
Pol_CR_12	188–198	304–314	11	0.9771
Pol_CR_13	207–221	323–337	15	0.9770
Pol_CR_14	249–259	367–382	11	0.9666
Pol_CR_15	279–301	402–424	23	0.9744
Pol_CR_16	303–308	426–431	6	0.9695
Pol_CR_17	316–335	440–459	20	0.9739
Pol_CR_18	362–367	486–491	6	0.9729
Pol_CR_19	369–374	493–498	6	0.9690
Pol_CR_20	400–408	524–532	9	0.9720
Pol_CR_21	411–419	535–543	9	0.9700
Pol_CR_22	425–429	549–553	5	0.9640
Pol_CR_23	469–476	593–600	8	0.9762
Pol_CR_24	478–494	602–618	17	0.9683
Pol_CR_25	505–509	629–633	5	0.9769
Pol_CR_26	517–521	641–645	5	0.9679
Pol_CR_27	536–540	660–664	5	0.9726
Pol_CR_28	548–553	672–677	6	0.9664
Pol_CR_29	557–575	681–699	19	0.9665
Pol_CR_30	599–611	723–735	13	0.9776
Pol_CR_31	613–617	737–741	5	0.9768
Pol_CR_32	628–634	752–758	7	0.9634
Pol_CR_33	650–654	774–778	5	0.9576
Pol_CR_34	657–662	781–786	6	0.9697
Pol_CR_35	675–683	799–807	9	0.9711
Pol_CR_36	688–695	812–819	8	0.9727
Pol_CR_37	699–707	823–831	9	0.9755
Pol_CR_38	709–720	833–844	12	0.9675
Pol_CR_39	738–742	862–866	5	0.9774
Pol_CR_40	751–757	875–881	7	0.9755
Pol_CR_41	761–779	885–903	19	0.9719
Pol_CR_42	781–786	905–910	6	0.9681
Pol_CR_43	792–796	916–920	5	0.9756
Pol_CR_44	801–811	925–935	11	0.9680
Pol_CR_45	818–824	942–948	7	0.9674
Pol_CR_46	847–857	971–981	11	0.9708
Pol_CR_47	859–874	983–998	16	0.9762
Pol_CR_48	881–888	1005–1012	8	0.9766
Pol_CR_49	894–901	1018–1025	8	0.9707

**Table 8:** S-index statistics for Env protein aligns.

Subtype	S_m_	CI−	CI+
All sequences	0.8320	0.8142	0.8487
A1	0.8754	0.8611	0.8900
A2	0.8716	0.8564	0.8858
A6	0.8886	0.8742	0.9013
B	0.9000	0.8872	0.9120
C	0.8731	0.8591	0.8867
D	0.8340	0.8177	0.8506
F1	0.8733	0.8582	0.8878
F2	0.8679	0.8529	0.8826
G	0.8466	0.8302	0.8619
H	0.8868	0.8723	0.9002
J	0.7989	0.7829	0.8126
K	0.8652	0.8497	0.8799

**Table 9:** Statistics of conserved regions detected in Env.

Subtype	Conserved Regions	Mean Conserved Region Length	S_m_ in Conserved Regions
All sequences	33	8.70	0.9666
A1	34	8.94	0.9885
A2	40	8.75	0.9746
A6	40	9.20	0.9815
B	41	9.15	0.9840
C	41	8.68	0.9794
D	34	8.18	0.9642
F1	32	8.88	0.9846
F2	39	8.08	0.9806
G	33	7.97	0.9749
H	37	8.54	0.9881
J	36	7.42	0.9192
K	31	8.48	0.9831

**Table 10:** Conserved region summary for Env polyprotein aligns.

Region Name	HXB2 Coordinates	Alignment Coordinates	Length	S_m_
Env_CR_1	34–44	36–46	11	0.9920
Env_CR_2	49–59	51–61	11	0.9635
Env_CR_3	65–78	67–80	14	0.9791
Env_CR_4	92–97	94–99	6	0.9478
Env_CR_5	106–119	108–121	14	0.9603
Env_CR_6	121–128	123–130	8	0.9752
Env_CR_7	202–206	272–276	5	0.9872
Env_CR_8	211–217	281–287	7	0.9846
Env_CR_9	223–227	293–297	5	0.9624
Env_CR_10	244–250	314–320	7	0.9882
Env_CR_11	252–266	322–336	15	0.9718
Env_CR_12	377–384	456–463	8	0.9603
Env_CR_13	417–425	519–527	9	0.9303
Env_CR_14	430–436	532–538	7	0.9540
Env_CR_15	474–486	584–596	13	0.9790
Env_CR_16	505–509	615–619	5	0.9542
Env_CR_17	518–531	630–643	14	0.9834
Env_CR_18	533–539	645–651	7	0.9601
Env_CR_19	541–549	653–661	9	0.9828
Env_CR_20	555–561	667–673	7	0.9774
Env_CR_21	565–579	677–691	15	0.9796
Env_CR_22	586–591	698–703	6	0.9559
Env_CR_23	593–598	705–710	6	0.9829
Env_CR_24	610–614	722–727	6	0.9378
Env_CR_25	675–679	788–792	5	0.9937
Env_CR_26	685–694	798–807	10	0.9584
Env_CR_27	703–713	816–826	11	0.9630
Env_CR_28	843–847	964–968	5	0.9748

**Table 11:** S-index statistics for Vif protein aligns.

Subtype	S_m_	CI−	CI+
All sequences	0.8809	0.8538	0.9075
A1	0.9183	0.8950	0.9372
A2	0.9132	0.8869	0.9366
A6	0.9322	0.9137	0.9497
B	0.9221	0.9013	0.9434
C	0.9081	0.8833	0.9299
D	0.8838	0.8577	0.9101
F1	0.8999	0.8760	0.9254
F2	0.9084	0.8808	0.9317
G	0.8795	0.8529	0.9070
H	0.9314	0.9119	0.9503
J	0.9010	0.8735	0.9251
K	0.8982	0.8700	0.9239

**Table 12:** Statistics of conserved regions detected in Vif.

Subtype	Conserved Regions	Mean Conserved Region Length	S_m_ in Conserved Regions
All sequences	7	7.00	0.9832
A1	10	7.40	0.9941
A2	9	7.22	1.0000
A6	8	7.13	0.9900
B	8	7.88	0.9930
C	10	6.40	0.9913
D	8	7.25	0.9794
F1	8	7.38	0.9955
F2	8	7.00	1.0000
G	8	6.38	0.9854
H	7	5.86	0.9958
J	10	7.60	0.9785
K	5	7.00	1.0000

**Table 13:** Conserved region summary for Vif polyprotein aligns.

Region Name	HXB2 Coordinates	Alignment Coordinates	Length	S_m_
VIf_CR_1	1–16	1–16	16	0.9761
VIf_CR_2	52–59	53–60	8	0.9738
VIf_CR_3	68–72	70–74	5	0.9923
VIf_CR_4	141–150	146–155	10	0.9777
VIf_CR_5	161–166	166–171	6	0.9877
VIf_CR_6	171–175	176–180	5	0.9906

**Table 14:** S-index statistics for Vpr protein aligns.

Subtype	S_m_	CI−	CI+
All sequences	0.9091	0.8680	0.9441
A1	0.9378	0.9068	0.9649
A2	0.9026	0.8602	0.9398
A6	0.9434	0.9134	0.9697
B	0.9400	0.9079	0.9663
C	0.9290	0.8976	0.9554
D	0.9093	0.8655	0.9447
F1	0.9336	0.9058	0.9589
F2	0.9070	0.8697	0.9429
G	0.9063	0.8649	0.9403
H	0.9442	0.9166	0.9666
J	0.9100	0.8742	0.9442
K	0.9024	0.8641	0.9392

**Table 15:** Statistics of conserved regions detected in Vpr.

Subtype	Conserved Regions	Mean Conserved Region Length	S_m_ in Conserved Regions
All sequences	4	6.50	0.9843
A1	5	6.40	0.9946
A2	4	5.25	1.0000
A6	6	5.66	0.9929
B	5	5.40	0.9937
C	5	6.20	0.9935
D	3	6.00	0.9854
F1	4	6.25	0.9932
F2	4	6.50	1.0000
G	5	6.00	0.9892
H	6	6.50	0.9940
J	4	7.00	1.0000
K	3	6.33	1.0000

**Table 16:** Conserved region summary for Vpr polyprotein aligns.

Region Name	HXB2 Coordinates	Alignment Coordinates	Length	S_m_
Vpr_CR_1	7–12	7–12	6	0.9749
Vpr_CR_2	29–36	29–36	8	0.9891
Vpr_CR_3	49–54	49–54	6	0.9864
Vpr_CR_4		78–83	6	0.9867

**Table 17:** S-index statistics for Rev protein aligns.

Subtype	S_m_	CI−	CI+
All sequences	0.8246	0.7866	0.8613
A1	0.8713	0.8358	0.9057
A2	0.8622	0.8260	0.8968
A6	0.9064	0.8826	0.9306
B	0.9047	0.8750	0.9320
C	0.8562	0.8187	0.8868
D	0.8365	0.8018	0.8696
F1	0.8751	0.8384	0.9079
F2	0.8772	0.8421	0.9111
G	0.8408	0.8026	0.8778
H	0.9295	0.9092	0.9493
J	0.8524	0.8161	0.8871
K	0.8610	0.8287	0.8932

**Table 18:** Statistics of conserved regions detected in Rev.

Subtype	Conserved Regions	Mean Conserved Region Length	S_m_ in Conserved Regions
All sequences	4	6.50	0.9694
A1	4	6.00	0.9919
A2	1	5.00	1.0000
A6	2	5.50	0.9928
B	4	7.25	0.9864
C	4	6.50	0.9877
D	5	5.80	0.9702
F1	5	9.20	0.9872
F2	4	6.00	1.0000
G	5	6.20	0.9715
H	5	8.40	1.0000
J	2	9.00	0.9905
K	5	6.60	0.9603

**Table 19:** Conserved region summary for Rev polyprotein aligns.

Region Name	HXB2 Coordinates	Alignment Coordinates	Length	S_m_
Rev_CR_1	1–6	1–6	6	0.9877
Rev_CR_2	22–27	22–27	6	0.9711
Rev_CR_3	33–39	33–39	7	0.9466
Rev_CR_4	41–47	41–47	7	0.9721

**Table 20:** S-index statistics for Tat protein aligns.

Subtype	S_m_	CI−	CI+
All sequences	0.8205	0.7731	0.8662
A1	0.8752	0.8389	0.9083
A2	0.8532	0.8128	0.8922
A6	0.9045	0.8728	0.9330
B	0.8912	0.8560	0.9208
C	0.8726	0.8358	0.9102
D	0.8179	0.7730	0.8641
F1	0.8675	0.8273	0.9058
F2	0.8414	0.7996	0.8810
G	0.8285	0.7876	0.8695
H	0.9045	0.8724	0.9327
J	0.8539	0.8068	0.8912
K	0.8413	0.8002	0.8797

**Table 21:** Statistics of conserved regions detected in Tat.

Subtype	Conserved Regions	Mean Conserved Region Length	S_m_ in Conserved Regions
All sequences	3	7.66	0.9659
A1	4	5.75	0.9922
A2	3	5.66	1.0000
A6	2	11.50	0.9923
B	3	8.66	0.9821
C	2	6.00	0.9910
D	3	5.33	0.9729
F1	5	5.60	0.9770
F2	2	8.50	0.9849
G	2	8.50	0.9631
H	3	5.33	0.9917
J	3	6.66	0.9855
K	2	7.50	0.9907

**Table 22:** Conserved region summary for Tat polyprotein aligns.

Region Name	HXB2 Coordinates	Alignment Coordinates	Length	S_m_
Tat_CR_1	13–18	13–18	6	0.9807
Tat_CR_2	41–52	41–52	12	0.9676

**Table 23:** S-index statistics for Nef protein aligns.

Subtype	S_m_	CI−	CI+
All sequences	0.8662	0.8381	0.8936
A1	0.9016	0.8773	0.9254
A2	0.8601	0.8365	0.8837
A6	0.9263	0.9058	0.9442
B	0.9047	0.8825	0.9274
C	0.8920	0.8694	0.9156
D	0.8562	0.8292	0.8826
F1	0.8911	0.8688	0.9127
F2	0.8689	0.8436	0.8926
G	0.8548	0.8267	0.8806
H	0.9117	0.8890	0.9317
J	0.8511	0.8228	0.8766
K	0.8474	0.8219	0.8716

**Table 24:** Statistics of conserved regions detected in Nef.

Subtype	Conserved Regions	Mean Conserved Region Length	S_m_ in Conserved Regions
All sequences	8	7.25	0.9755
A1	9	8.22	0.9889
A2	7	9.28	0.9736
A6	10	7.50	0.9895
B	10	7.10	0.9836
C	7	8.00	0.9823
D	8	6.88	0.9657
F1	11	7.00	0.9754
F2	7	9.71	0.9705
G	7	8.42	0.9700
H	7	7.00	0.9940
J	10	7.00	0.9747
K	7	7.57	0.9797

**Table 25:** Conserved region summary for Nef polyprotein aligns.

Region Name	HXB2 Coordinates	Alignment Coordinates	Length	S_m_
Nef_CR_1	1–7	1–7	8	0.9654
Nef_CR_2	66–70	94–98	5	0.9885
Nef_CR_3	72–80	100–108	9	0.9893
Nef_CR_4	90–97	118–125	8	0.9887
Nef_CR_5	109–115	137–143	7	0.9784
Nef_CR_6		154–160	7	0.9745

**Table 26:** S-index statistics for Vpu protein aligns.

Genotype	S_m_	CI−	CI+
All sequences	0.7839	0.7307	0.8321
A1	0.8602	0.8155	0.9004
A2	0.8446	0.8025	0.8844
A6	0.8861	0.8448	0.9221
B	0.9046	0.8722	0.9346
C	0.8327	0.7889	0.8733
D	0.7975	0.7504	0.8457
F1	0.8412	0.7982	0.8820
F2	0.8167	0.7654	0.8665
G	0.8029	0.7593	0.8437
H	0.8479	0.8069	0.8862
J	0.8025	0.7604	0.8412
K	0.7725	0.7232	0.8194

**Table 27:** Statistics of conserved regions detected in Vpu.

Genotype	Conserved Regions	Mean Conserved Region Length	S_m_ in Conserved Regions
All sequences	1	10.00	0.9541
A1	4	5.00	0.9873
A2	1	5.00	1.0000
A6	3	9.33	0.9799
B	3	7.33	0.9778
C	2	6.00	0.9687
D	2	8.00	0.9687
F1	2	8.00	0.9642
F2	1	9.00	0.9766
G	2	8.00	0.9553
H	2	7.50	0.9783
J	3	7.33	0.9315
K	4	6.25	0.9270

**Table 28:** Number of analyzed sequences by region, protein, and genotype.

Subtype	Env	Gag	Nef	Pol	Rev	Tat	Vpu
A1	111	105	106	106	105	105	106
A2	8	9	8	9	9	9	8
A6	193	196	196	194	194	195	195
B	199	200	199	196	197	200	200
C	190	197	196	189	191	201	200
D	93	98	97	99	95	93	95
F1	61	62	61	56	55	60	61
F2	11	13	10	12	9	13	10
G	89	91	93	96	96	96	92
H	100	98	97	99	10	98	96
J	16	22	10	16	16	16	16
K	12	12	12	16	11	11	11
All subtypes	1083	1103	1085	1088	988	1097	1090

## Data Availability

The original contributions presented in this study are included in the article. Further inquiries can be directed to the corresponding author.

## Abbreviations

The following abbreviations are used in this manuscript:

HIV	human immunodeficiency virus
WHO	World Health Organization
CRF	circulating recombinant form
ART	antiretroviral therapy
NGS	next-generation sequencing
UTR	untranslated region
GLS	Gag leader sequence
CI	confidence interval
Gag, pr55	group-specific antigen
CR	conserved region
Pol	polymerase
Env, gp160	envelope
Vif	viral infectivity factor
Vpr	viral protein R
Rev	regulator of expression
Tat	trans-activator of transcription
Nef	negative regulatory factor
Vpu	viral protein U
CA	capsid domain
MA	matrix domain
NC	nucleocapsid domain
SP	spacer peptide
HBR	highly basic region
NTD	N-terminal domain
CTD	C-terminal domain
CypA	cyclophilin A
MHR	major homology region
PIC	preintegration complex
ZF	zinc finger
PR	protease
RT	reverse transcriptase
IN	integrase
RT_Rtv	retroviral reverse transcriptase
RNase_H	retroviral ribonuclease H
RVT_thumb	RT thumb domain
RVT_connect	reverse transcriptase connection domain
PPT	polypurine tract
rve	retroviral-like integrase
IN_DBD_C	integrase DNA binding domain
IN_Zn	integrase zinc binding domain
RRE	rev response element
ARM	arginine-rich motif
